# Supplementary material for: Endothelial sensitivity to pro-fibrotic signals links systemic exposure to pulmonary fibrosis
Source: Cell Death Dis. 2025 Jul 7;16(1):500. doi: 10.1038/s41419-025-07824-5 (PMC12234957; doi:10.1038/s41419-025-07824-5)
Supplement: Supplementary file 1 — Supplementary information [file 41419_2025_7824_MOESM1_ESM.pdf]

Supplementary information

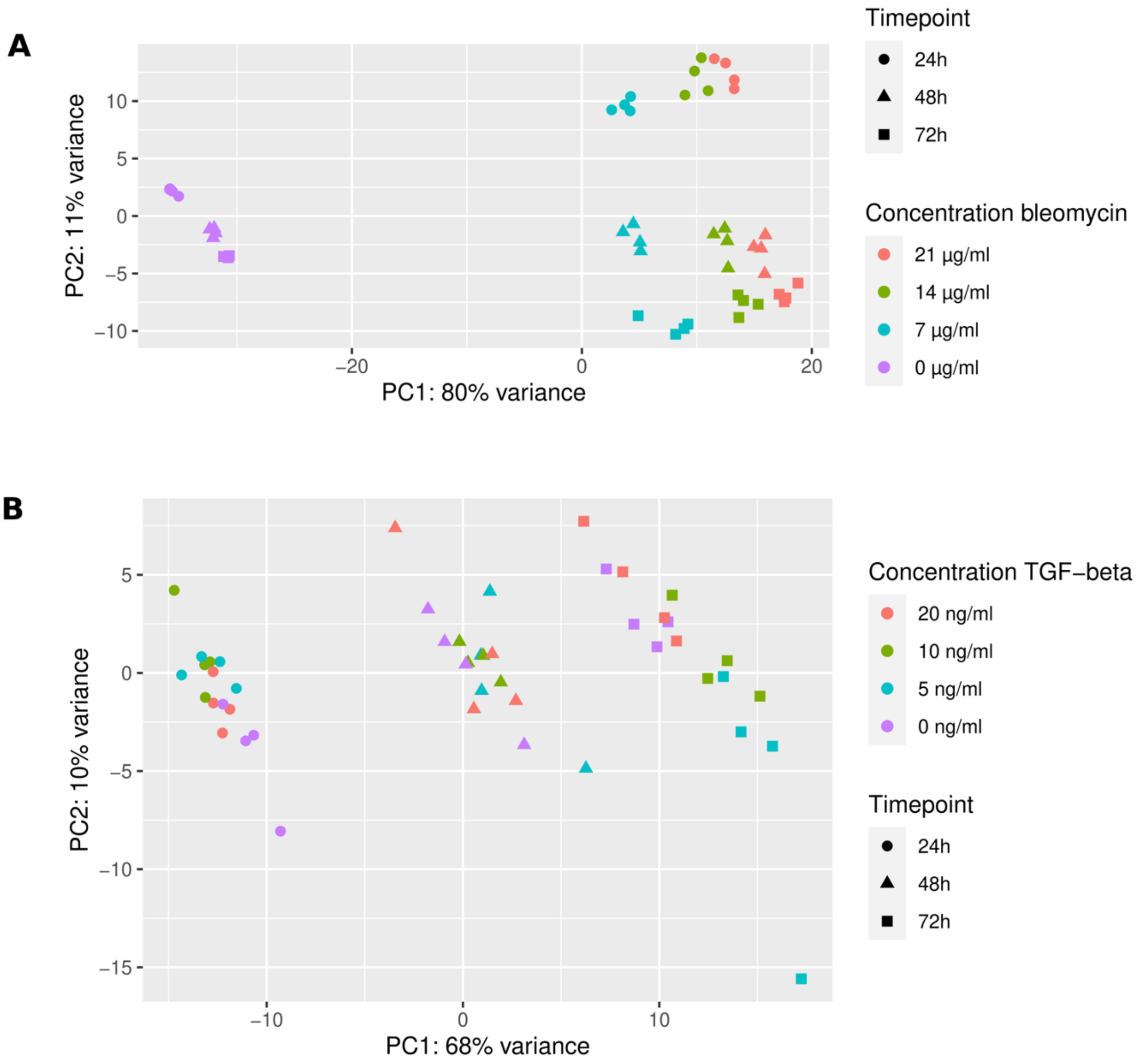

Figure S1: **Principal component analysis (PCA) of datasets. A.** PCA of the bleomycin dataset based on variance-stabilisation transformed (vst) counts of the top 1,000 variable genes. **B**. PCA of the TGF-beta dataset based on vst counts of the top 1,000 variable genes.

A

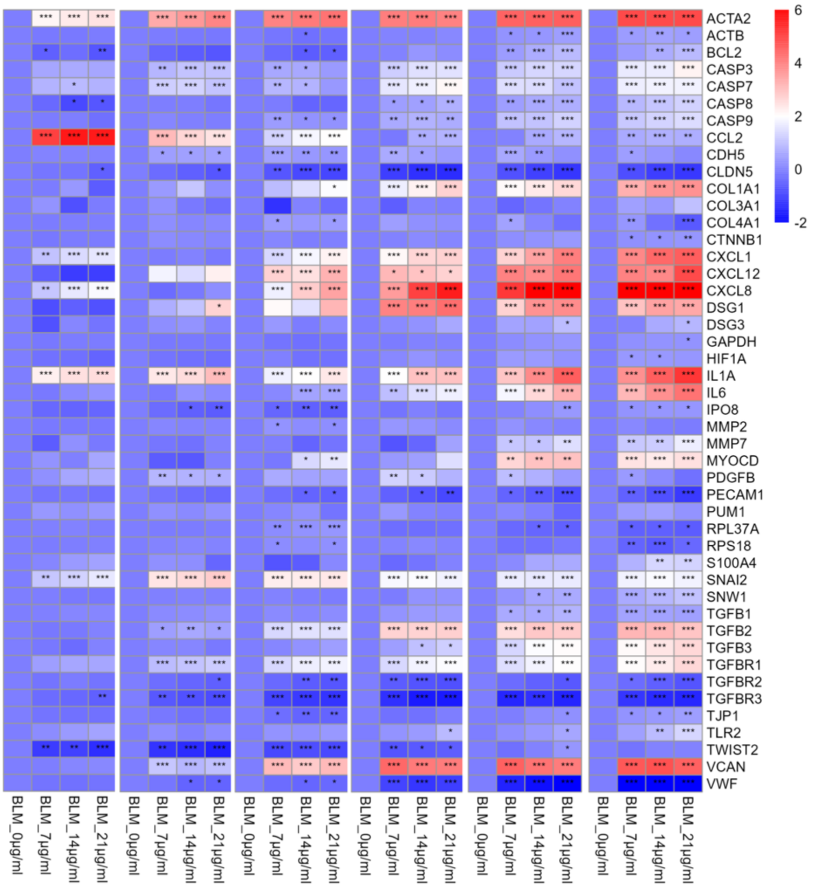
6
4
2
0
-2
ACTA2
ACTB
BCL2
CASP3
CASP7
CASP8
CASP9
CCL2
CDH5
CLDN5
COL1A1
COL3A1
COL4A1
CTNNB1
CXCL1
CXCL12
CXCL8
DSG1
DSG3
GAPDH
HIF1A
IL1A
IL6
IPO8
MMP2
MMP7
MYOCD
PDGFB
PECAM1
PUM1
RPL37A
RPS18
S100A4
SNAI2
SNW1
TGFB1
TGFB2
TGFB3
TGFBR1
TGFBR2
TGFBR3
TJP1
TLR2
TWIST2
VCAN
VWF
BLM_0µg/ml
BLM_7µg/ml
BLM_14µg/ml
BLM_21µg/ml

B

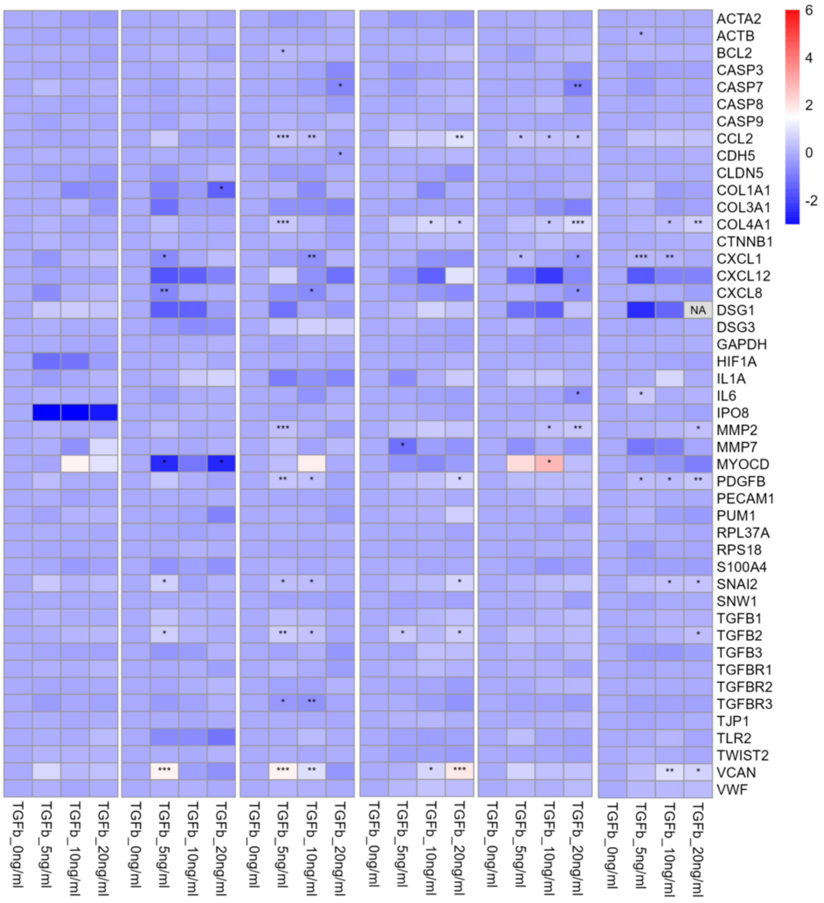
6
4
2
0
-2
NA
ACTA2
ACTB
BCL2
CASP3
CASP7
CASP8
CASP9
CCL2
CDH5
CLDN5
COL1A1
COL3A1
COL4A1
CTNNB1
CXCL1
CXCL12
CXCL8
DSG1
DSG3
GAPDH
HIF1A
IL1A
IL6
IPO8
MMP2
MMP7
MYOCD
PDGFB
PECAM1
PUM1
RPL37A
RPS18
S100A4
SNAI2
SNW1
TGFB1
TGFB2
TGFB3
TGFBR1
TGFBR2
TGFBR3
TJP1
TLR2
TWIST2
VCAN
VWF
TGFb_0ng/ml
TGFb_5ng/ml
TGFb_10ng/ml
TGFb_20ng/ml

Figure S2: High-throughput qPCR with extended sampling timepoints. A. Bleomycin. **B.** TGF-beta. Heatmaps shows relative gene expression fold change compared to control condition (0ng/ml, 0µg/ml) at each timepoint. Statistical significance is calculated for each timepoint separately: * equals p<0.05, ** equals p<0.01, *** equals p<0.001.

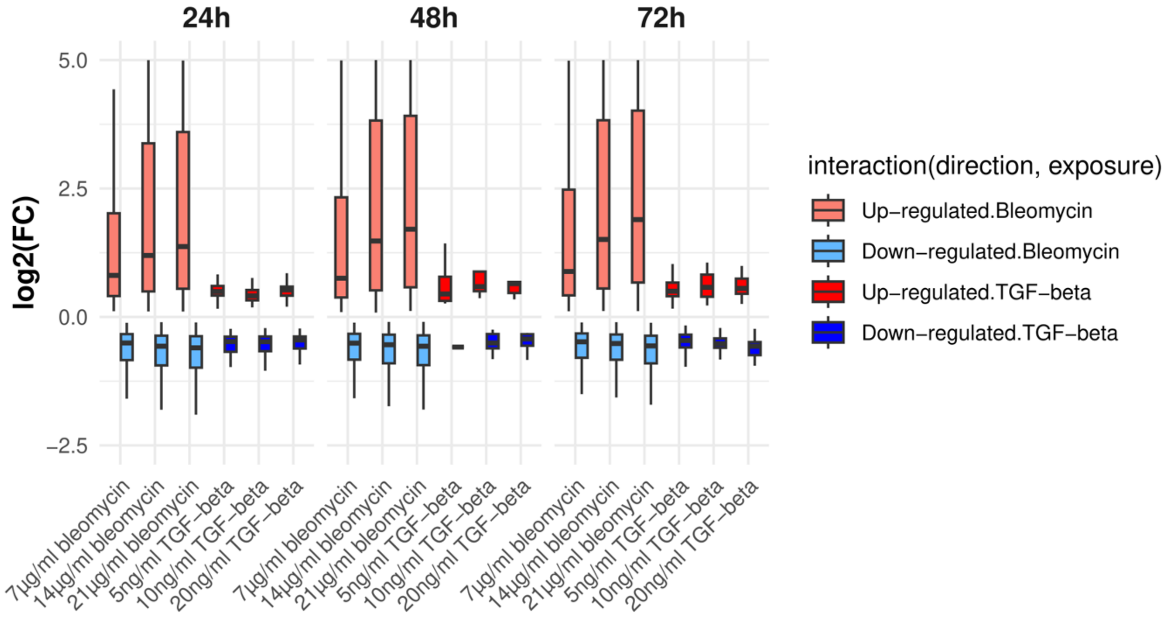

Figure S3: **Magnitude of change in gene expression under both exposures.** Fold changes on log_2 scale for all genes differentially expressed for each pairwise comparison of an experimental condition (concentration + timepoint) versus controls for both datasets. Up- and downregulated genes are plotted separately.

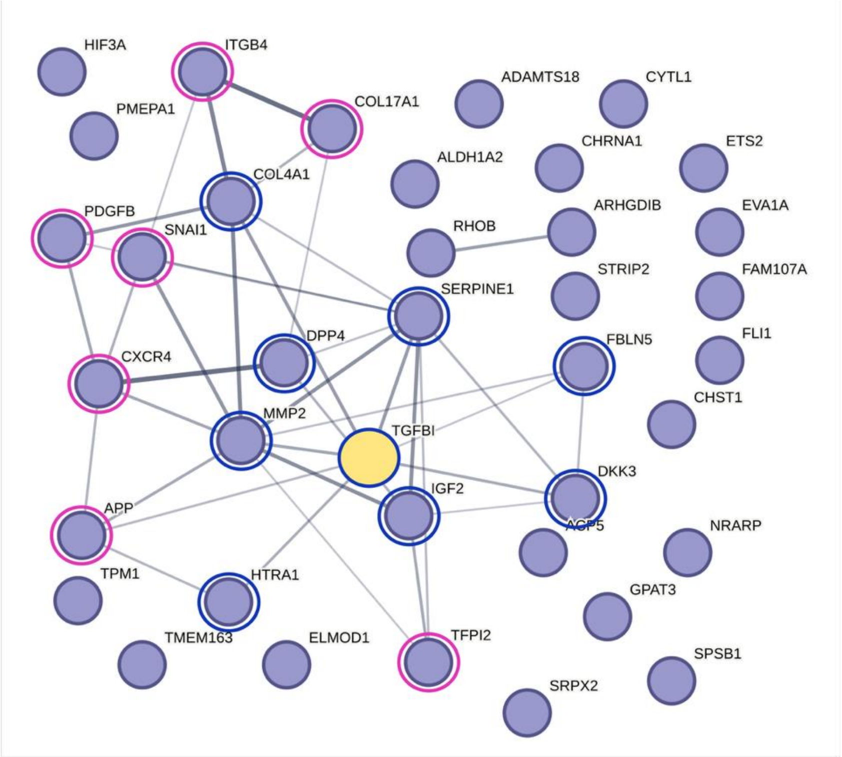

Figure S4: **Gene expression alterations under TGF-beta.** Protein-protein interaction network derived from the STRING database containing proteins whose gene expression was dose-dependently altered by TGF-beta exposure at all measured timepoints. Proteins marked with a blue circle are direct interaction partners of TGFB1 (yellow), proteins marked with red circles are indirect interaction partners via one other direct neighbour of TGFB1.

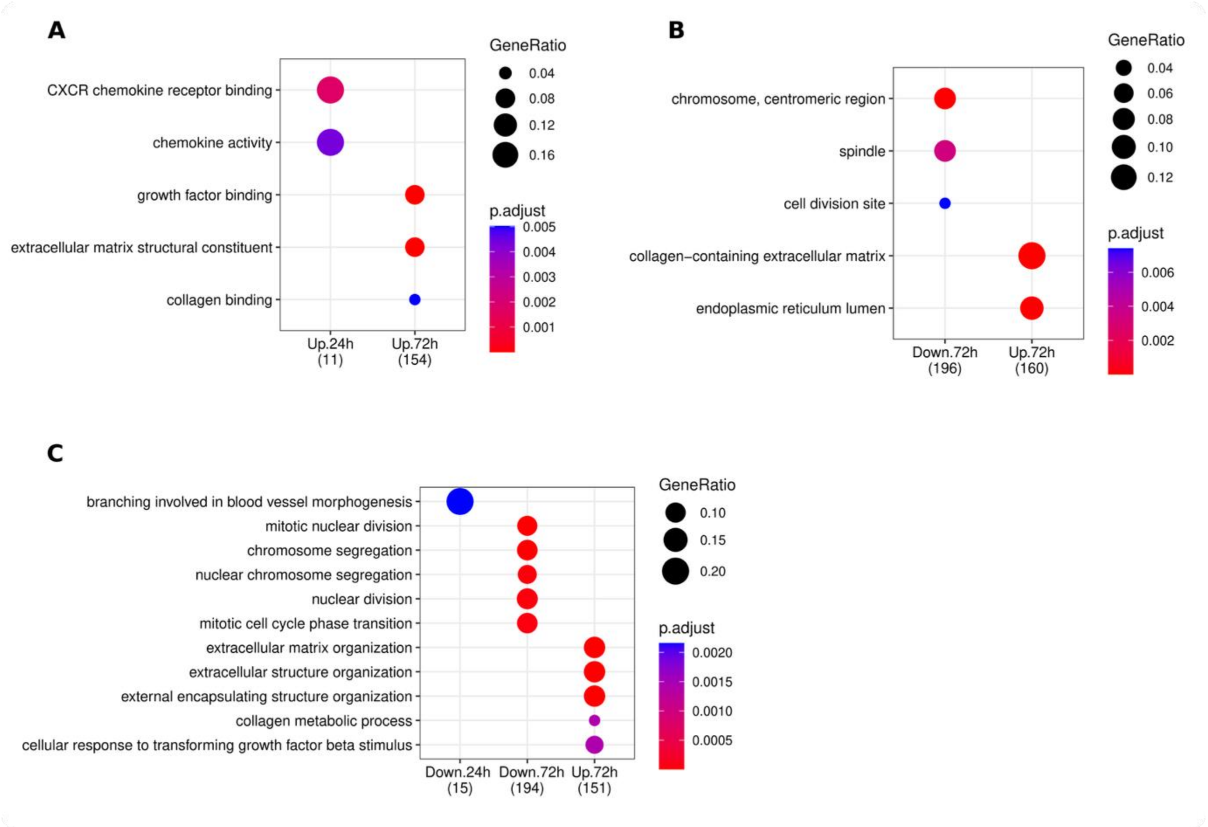

Figure S5: Intersection of genes differentially expressed under bleomycin and TGF-beta. Differentially expressed genes per timepoint were split into up- and downregulated genes and intersected between bleomycin and TGF-beta exposure. Overrepresentation test was performed against the gene sets of Gene Ontology (GO). **A.** Enriched GO molecular function gene sets. **B.** Enriched GO cellular component gene sets. **C.** Enriched GO biological process gene sets. The dot size indicates the gene ratio *i.e.*, the ratio of identified genes to genes of the gene set. The colour code indicates the Benjamini-Hochberg-adjusted p-value of the Fisher's exact test. The top five enriched terms are plotted.

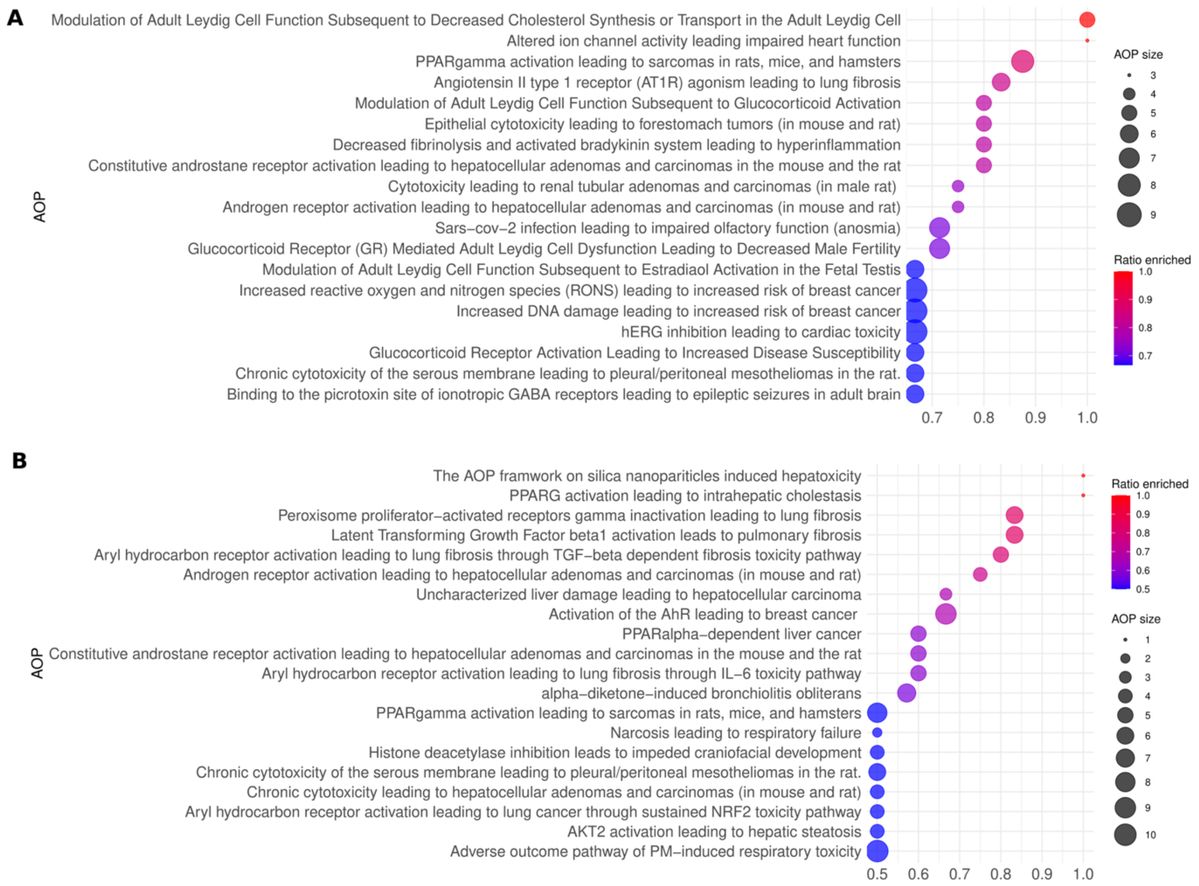

Figure S6: AOPs enriched by dysregulated genes upon exposure. A. AOPs with enriched KEs by dysregulated genes under bleomycin exposure. Genes that were both dose-dependent <u>and </u>differentially expressed (adjusted p-value < 0.01 and absolute LFC > 1) at any timepoint of the exposure and at any concentration were considered for the KE enrichment analysis (n = 6,833 genes). **B.** AOPs with enriched KEs by dysregulated genes under TGF-beta exposure. Genes that were both dose-dependent <u>and</u> differentially expressed (adjusted p-value < 0.01) at any timepoint of the exposure and at any concentration were considered for the KE enrichment analysis (n = 281 genes). **A + B.** The AOPs with the top 15 fractions enriched are plotted. Since multiple AOPs showed the same fraction of enriched KEs, the number of plotted AOPs is not equal 15. Colour code represents fraction of enriched KEs. Dot size represents total size of an AOP *i.e.*, number of KEs.

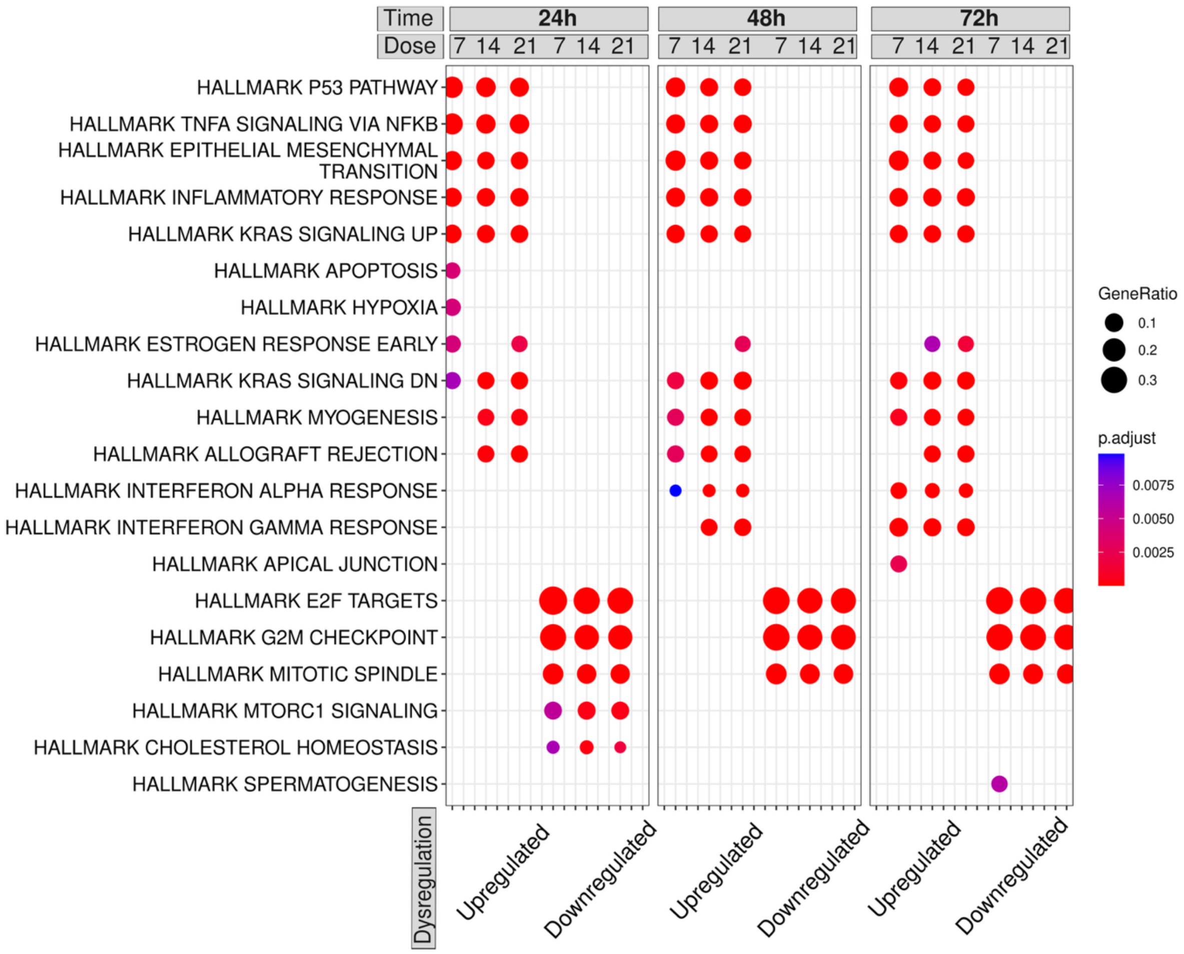

Figure S7: Hallmark pathways enriched under bleomycin. Functional analysis of up- and downregulated genes under bleomycin exposure. Overrepresentation test with dysregulated genes (adjusted p-value < 0.01 and absolute LFC > 1) against the gene sets of MSigDb Hallmark pathways. The dot size indicates the gene ratio *i.e.*, the ratio of identified genes to genes of the pathway. The colour code indicates the Benjamini-Hochberg-adjusted p-value of the Fisher's exact test. The top five enriched pathways are plotted, and already existing pathways are filled even if they are not among the top five pathways.

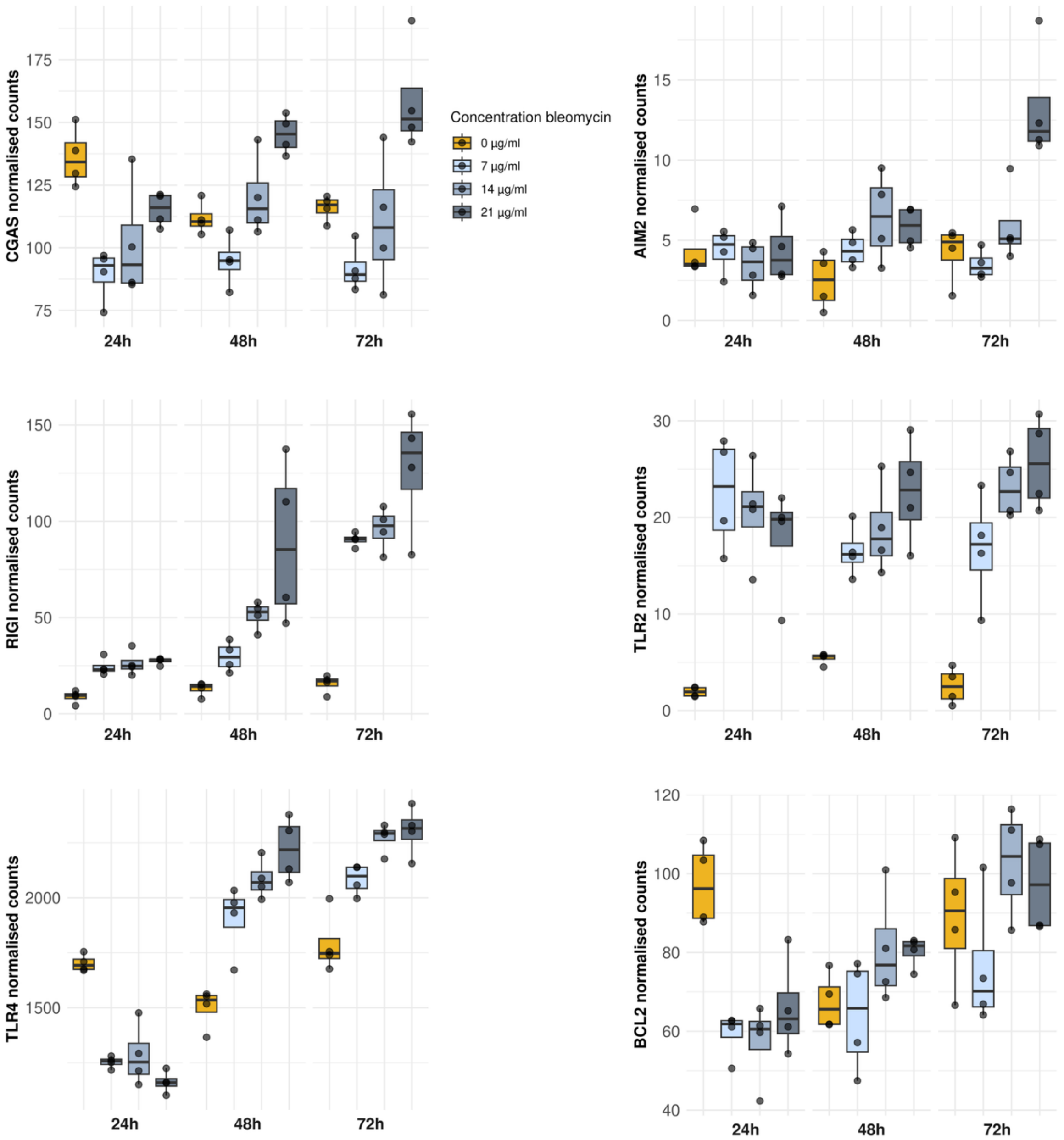

Figure S8: DAMP-sensing related genes and *BCL2* under bleomycin. Expression of DAMP-sensing related genes as well as *BCL2* for the different experimental conditions of bleomycin exposure. Plotted are the size-factor normalised RNA-Seq counts.

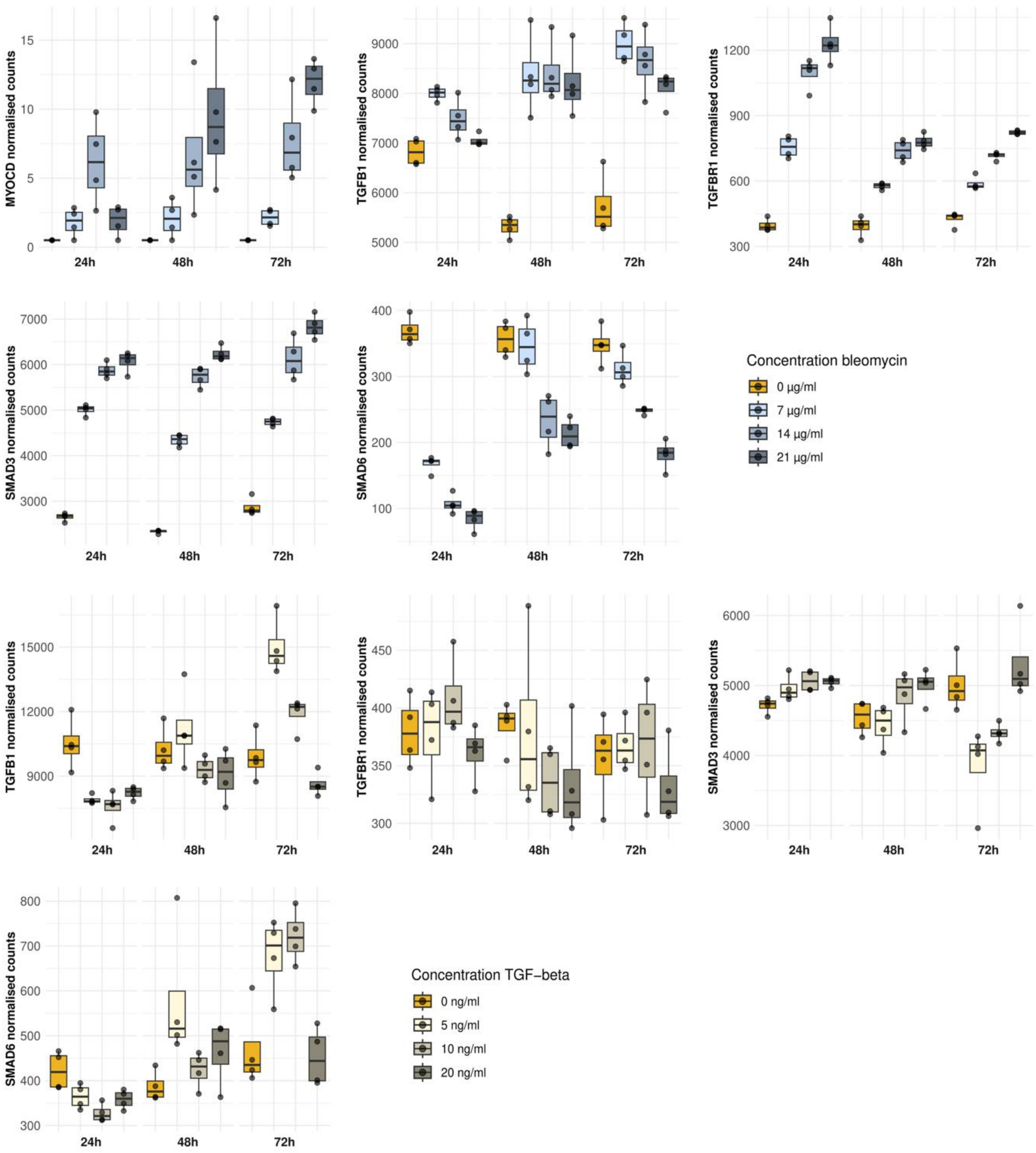

Figure S9: EndMT related genes under bleomycin and TGF-beta. Expression of *MYOCD* under bleomycin exposure, and expression of TGF-beta signalling-related genes for the different experimental conditions of bleomycin and TGF-beta exposure, respectively. Plotted are the size-factor normalised RNA-Seq counts.

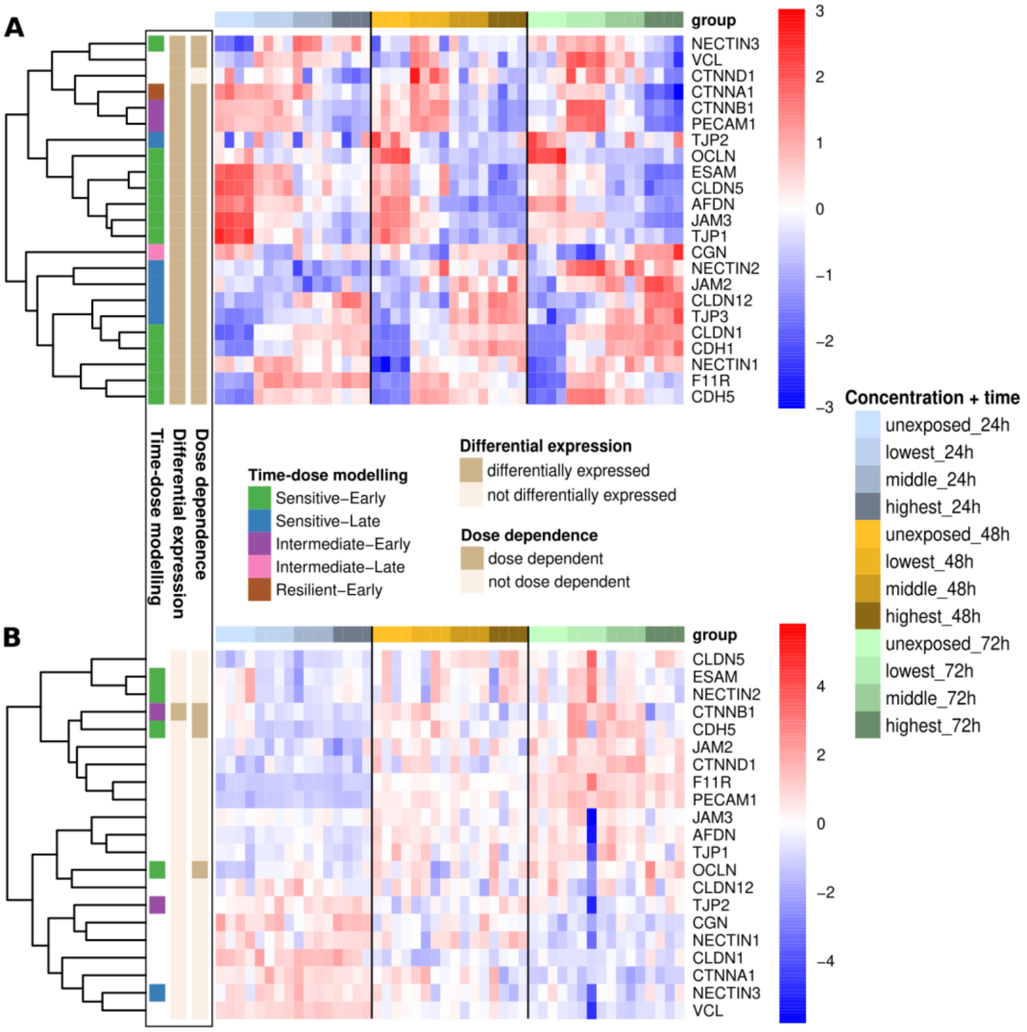

Figure S10: Cell-cell-contact related genes under bleomycin and TGF-beta. A. Expression of cell-cell contact related genes for the different experimental conditions of bleomycin exposure. **B.** Expression of cell-cell contact related genes for the different experimental conditions of TGF-beta exposure. The colour annotation of columns indicates the experimental condition, the row annotations refer to the label of the time-dose modelling, and the results of the differential expression and dose-dependent analysis. The heatmap colour code represents the row-wise z-score based on vst counts.

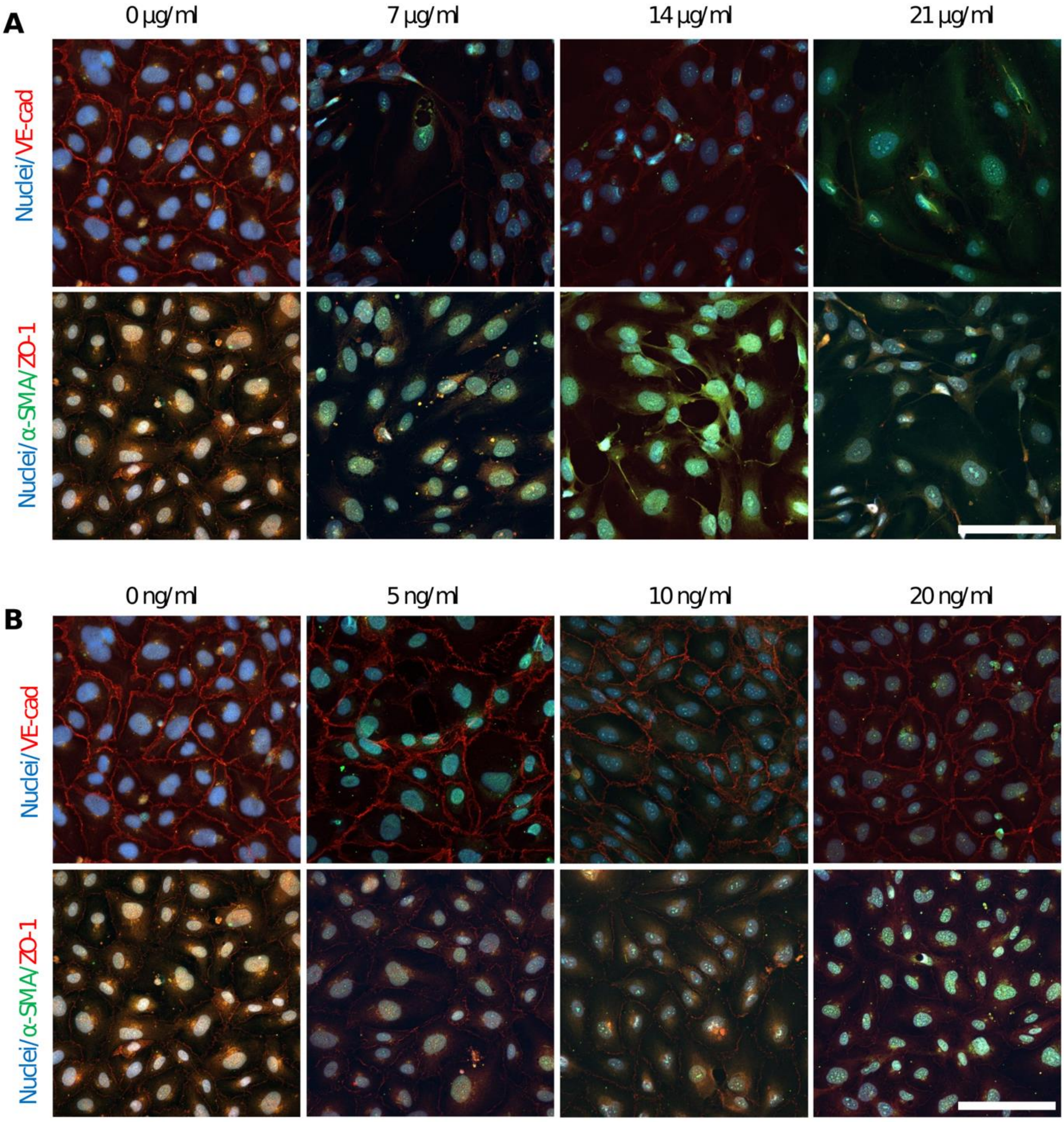

Figure S11: Immunocytochemical staining of HUVEC cells. Immunofluorescence staining from the cells exposed to bleomycin (**A**) or TGF-beta (**B**) for 72h. Cells exposed to 0, 7, 14, or 21 µg/ml bleomycin and 0, 5, 10, and 20 ng/ml TGF-beta, respectively, were stained against alpha-SMA as marker for mesenchymal lineages, VE-cadherin for endothelial specific adherens junctions, ZO-1 for tight junctions, and Hoechst 33342 for nuclei. Alpha-SMA is shown in green, VE-cadherin in red (top row), ZO-1 in red (lower row), and Hoechst 33342 in blue. Scale bar is 50 µm for all images. All images in line have been imaged with same settings (gain, exposure).

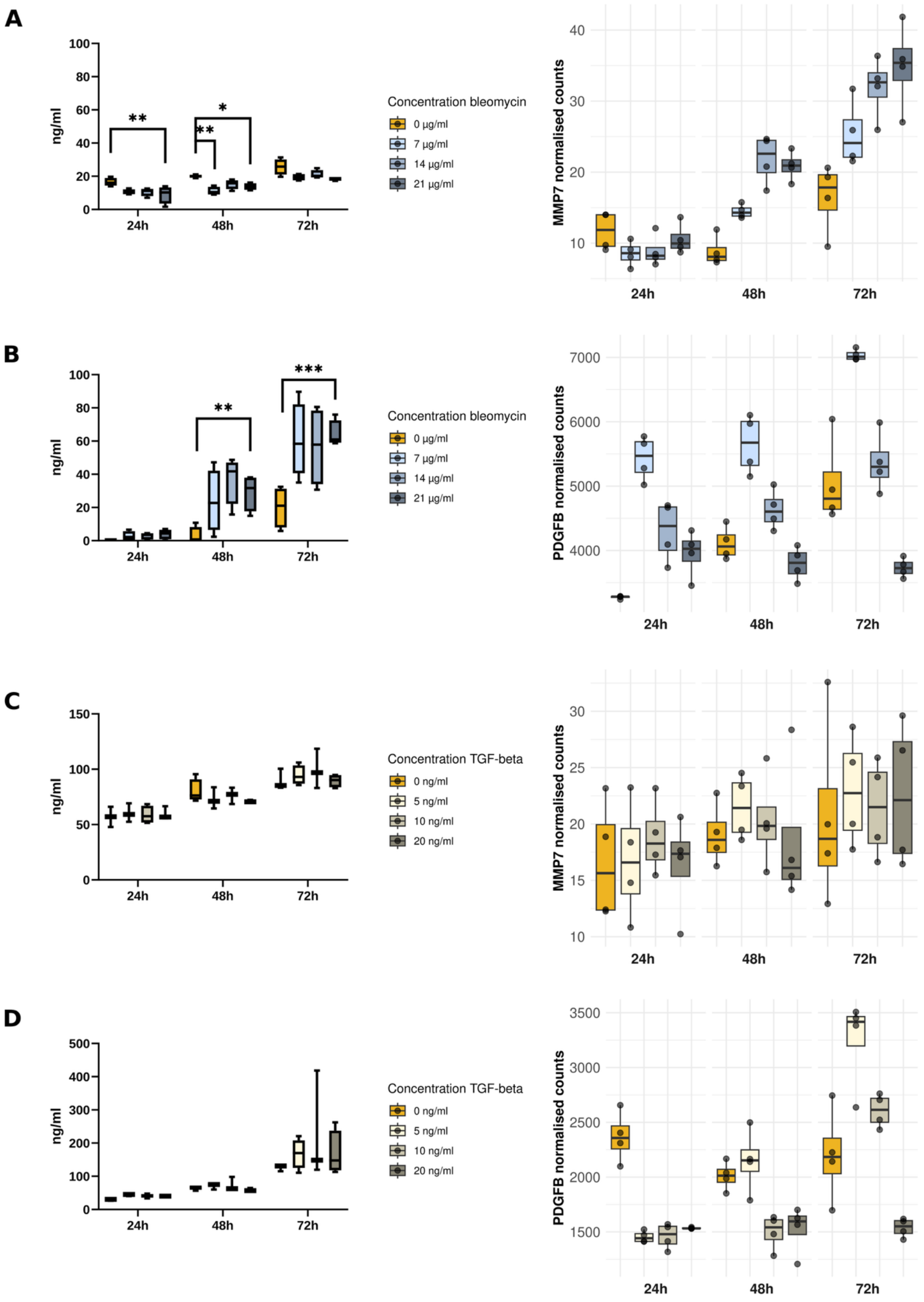

Figure S12: ECM-related protein secretion and gene expression under bleomycin and TGF-beta. A. MMP7 protein secretion (left) and *MMP7* gene expression (right) under bleomycin. **B.** PDGF-bb protein secretion (left) and *PDGFB* gene expression (right) under bleomycin. **C.** MMP7 protein secretion (left) and *MMP7* gene expression (right) under TGF-beta. **D.** PDGF-bb protein secretion (left) and *PDGFB* gene

expression (right) under TGF-beta. **A-D.** Plotted are the relative intensities from immunoassay (protein secretion) and the size-factor normalised RNA-Seq counts (gene expression). * equals p<0,05, ** equals p<0,01, *** equals p<0,001.

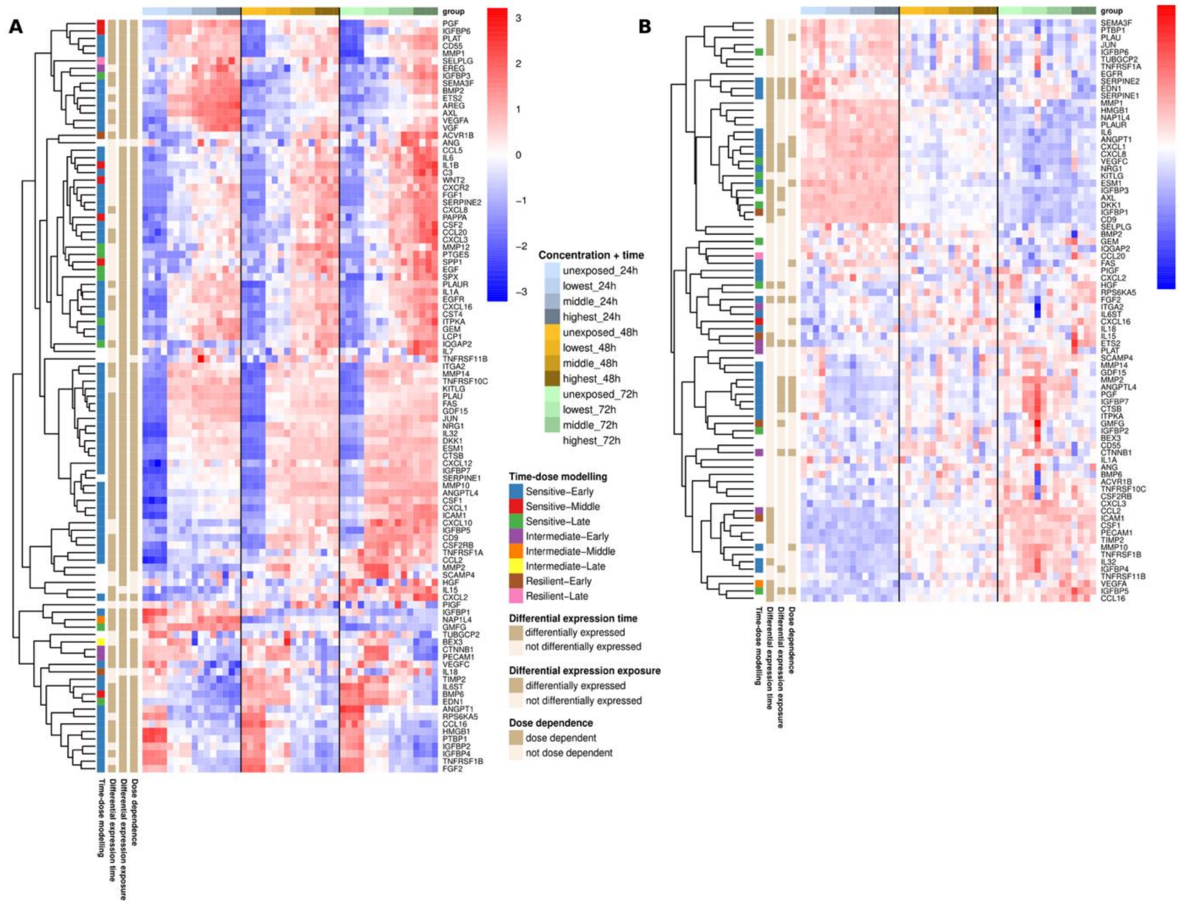

Figure S13: Cellular-senescence related genes under bleomycin and TGF-beta. A. Expression of the SenMayo cellular senescence gene set for the different experimental conditions of bleomycin exposure. **B.** Expression of the SenMayo cellular senescence gene set for the different experimental conditions of TGF-beta exposure. The colour annotation of columns indicates the experimental condition, the row annotations refer to the label of the time-dose modelling, and the results of the differential expression (tested for both variables time and exposure) and dose-dependent analysis. The heatmap colour code represents the row-wise z-score based on vst counts.

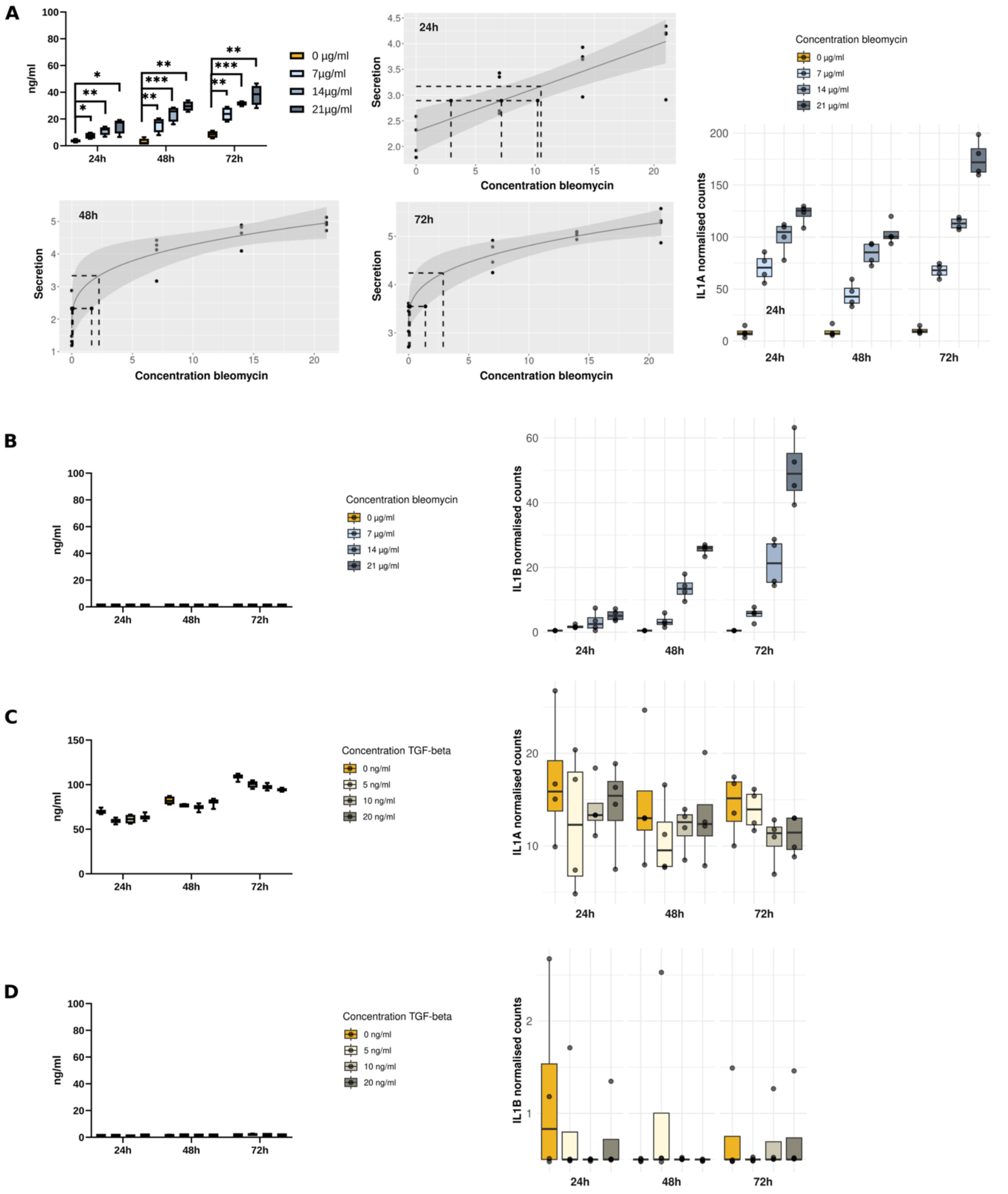

Figure S14: Interleukin-1 secretion and expression under bleomycin and TGF-beta. A. Top left: IL-1A protein secretion under bleomycin in relative intensities from the immunoassay. Middle: Optimal models of the dose-dependent analysis for IL-1A protein secretion under bleomycin for each timepoint. Plotted are the log_2-transformed relative intensities. Right: *IL1A* gene expression under bleomycin in size-factor normalised RNA-Seq counts. **B.** IL-1B protein secretion (left) and *IL1B* gene expression (right) under bleomycin. **C.** IL-1A protein secretion (left) and *IL1A* gene expression (right) under TGF-beta. **D.** IL-1B protein secretion (left) and *IL1B* gene expression (right) under TGF-beta. **A-D.** Plotted are the relative intensities from immunoassay (protein secretion) and the size-factor normalised RNA-Seq counts (gene expression). * equals p<0.05, ** equals p<0.01, *** equals p<0.001.

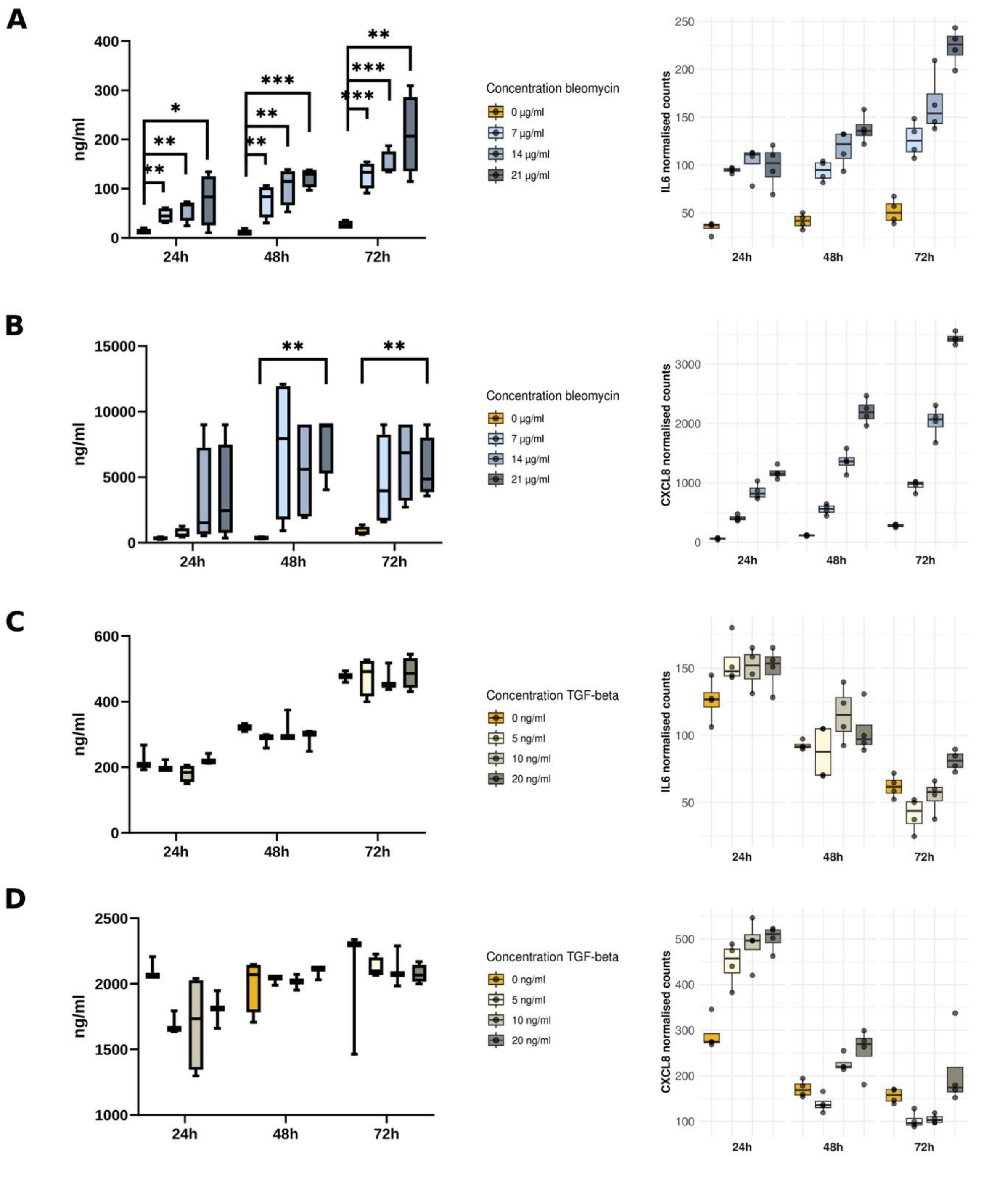

Figure S15: IL-6 and IL-8 protein secretion and gene expression under bleomycin and TGF-beta. A. IL-6 protein secretion (left) and *IL6* gene expression (right) under bleomycin. **B.** IL-8 protein secretion (left) and *CXCL8* gene expression (right) under bleomycin. **C.** IL-6 protein secretion (left) and *IL6* gene expression (right) under TGF-beta. **D.** IL-8 protein secretion (left) and *CXCL8* gene expression (right) under TGF-beta. **A-D.** Plotted are the relative intensities from immunoassay (protein secretion) and the size-factor normalised RNA-Seq counts (gene expression). * equals p<0.05, ** equals p<0.01, *** equals p<0.001.

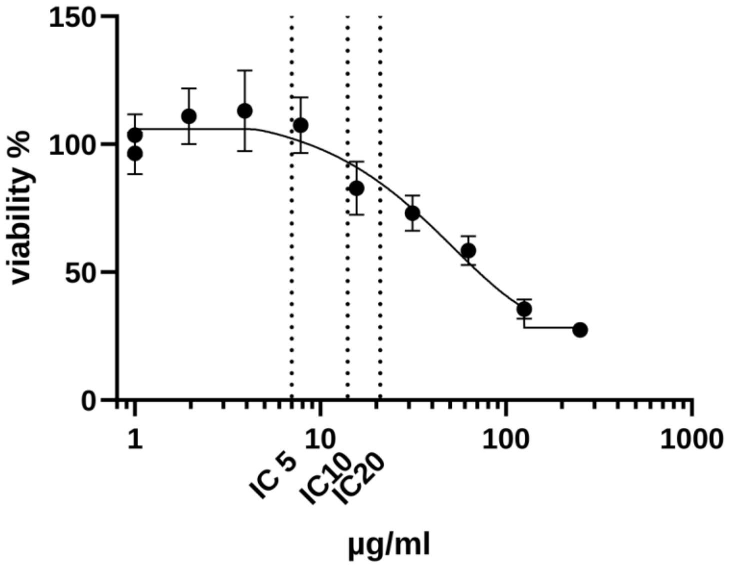

Figure S16: Viability of HUVEC cells under different doses of bleomycin. Viability of cells at selected doses within a dose range of bleomycin was tested with a Real time Glo MT Cell Viability Assay as described in the methods section. 7 µg/ml, 14 µg/ml, and 21µg/ml bleomycin were determined to correspond to IC5, IC10, and IC20, respectively, and used to investigate gene expression changes by RNA-Seq.

List of supplementary tables

Table S1. Excel file containing additional data. List of genes detected only under bleomycin exposure.

Table S2. Excel file containing additional data. Summary statistics of high-throughput qPCR for bleomycin exposure with additional timepoints.

Table S3. Excel file containing additional data. Summary statistics of high-throughput qPCR for TGF-beta exposure with additional timepoints.

Table S4. Excel file containing additional data. Differential expression analysis results bleomycin.

Table S5. Excel file containing additional data. Differential expression analysis results TGF-beta.

Table S6. Excel file containing additional data. Optimal models of the dose-dependent analysis for bleomycin and TGF-beta, respectively.

Table S7. Excel file containing additional data. Enriched Gene Ontology terms for the intersection of genes differentially expressed under bleomycin and TGF-beta, related to Figure S4.

Table S8. Excel file containing additional data. Enriched KEs for dysregulated genes by bleomycin and TGF-beta, respectively.

Table S9). Excel file containing additional data. Existing AOPs for PF and how their KEs were enriched by dysregulated genes by bleomycin and TGF-beta, respectively.

Table S10. Excel file containing additional data. Enriched KEGG and Hallmark pathways for dysregulated genes under bleomycin.

Table S11. Excel file containing additional data. Resulting gene labels of the time-dose modelling for bleomycin and TGF-beta, respectively.

Table S12. Excel file containing additional data. Summary statistics of high-throughput qPCR for TGF-beta exposure with additional doses.

Table S13. Excel file containing additional data. General FastQC statistics of the raw RNA-Seq data for the bleomycin and TGF-beta dataset, respectively.

Table S14. Excel file containing additional data. Gene panel used for the high-throughput qPCR.
